# Supplementary material for: Molecular model of TFIIH recruitment to the transcription-coupled repair machinery
Source: Nat Commun. 2025 Mar 8;16:2341. doi: 10.1038/s41467-025-57593-0 (PMC11890784; doi:10.1038/s41467-025-57593-0)
Supplement: Supplementary file 1 — Supplementary Information [file 41467_2025_57593_MOESM1_ESM.pdf]

SUPPLEMENTARY INFORMATION

SUPPLEMENTARY FIGURES

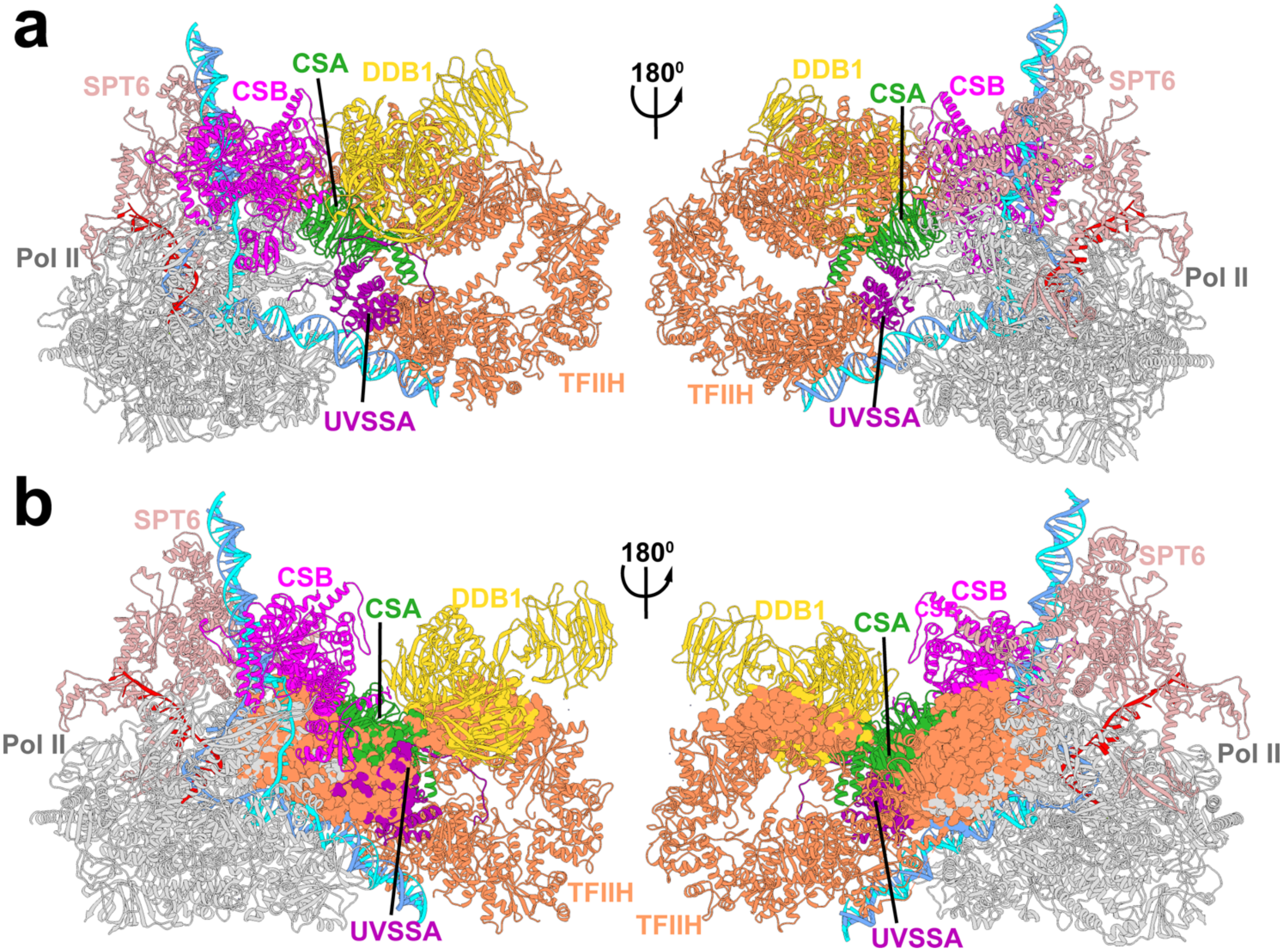

Supplementary Figure 1. Structure overlays show that the circular form of TFIIH clashing with core TCR proteins. Anterior and posterior views of the **a,** open- (PDB ID: 6O9L) and **b**, circular form of TFIIH (PDB ID: 6RO4) shown in cartoon representation overlayed onto the TCR–CSA–DDB1–VHS–ELOF1 cryo-EM structure (PDB ID: 8B3D). TFIIH is colored in orange, CSB in magenta, CSA in dark green, UVSSA in purple, DDB1 in gold, SPT6 in light brown, and Pol II in gray. Clashes between TFIIH residues and core TCR proteins – CSA, CSB, UVSSA and DDB1 – are shown in van-der-Waals representation to highlight incompatible regions.

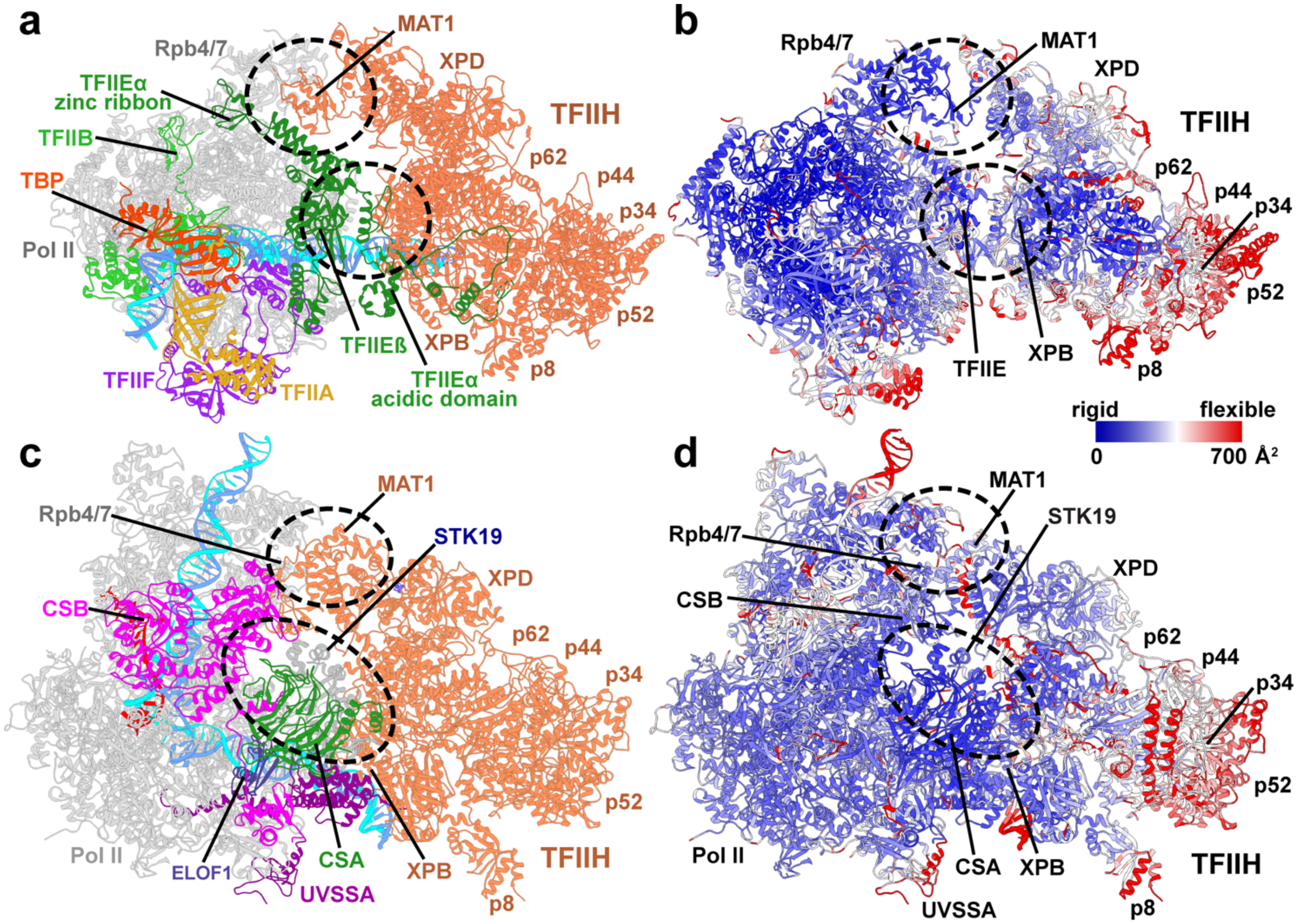

Supplementary Figure 2. Structural correspondence between our integrative model of the TCR–TFIIH assembly and the transcription pre-initiation complex. a, Structure of the human PIC in cartoon representation with TFIIH subunits and general transcription factors color-coded and labeled. **b,** Computed B-factors mapped onto the PIC structure and color-coded from red (high) to blue (low). **c,** Integrative structural model of the TCR-TFIIH complex with TFIIH subunits and core TCR factors color-coded and labeled. **d,** Computed B-factors mapped onto the TCR-TFIIH structure and color-coded from red (high) to blue (low). The key structural elements maintaining the integrity of the Pol II–TFIIH interface in both complexes are outlined with black dashed lines. The overall color-coding scheme is as follows: TFIIH is colored in orange, CSB in magenta, CSA in dark green, UVSSA in purple, ELOF1 in violet, and Pol II and STK19 in gray; TFIIA in gold, TBP in orange-red; TFIIB in light green, TFIIH in light purple; TFIIE in dark green.

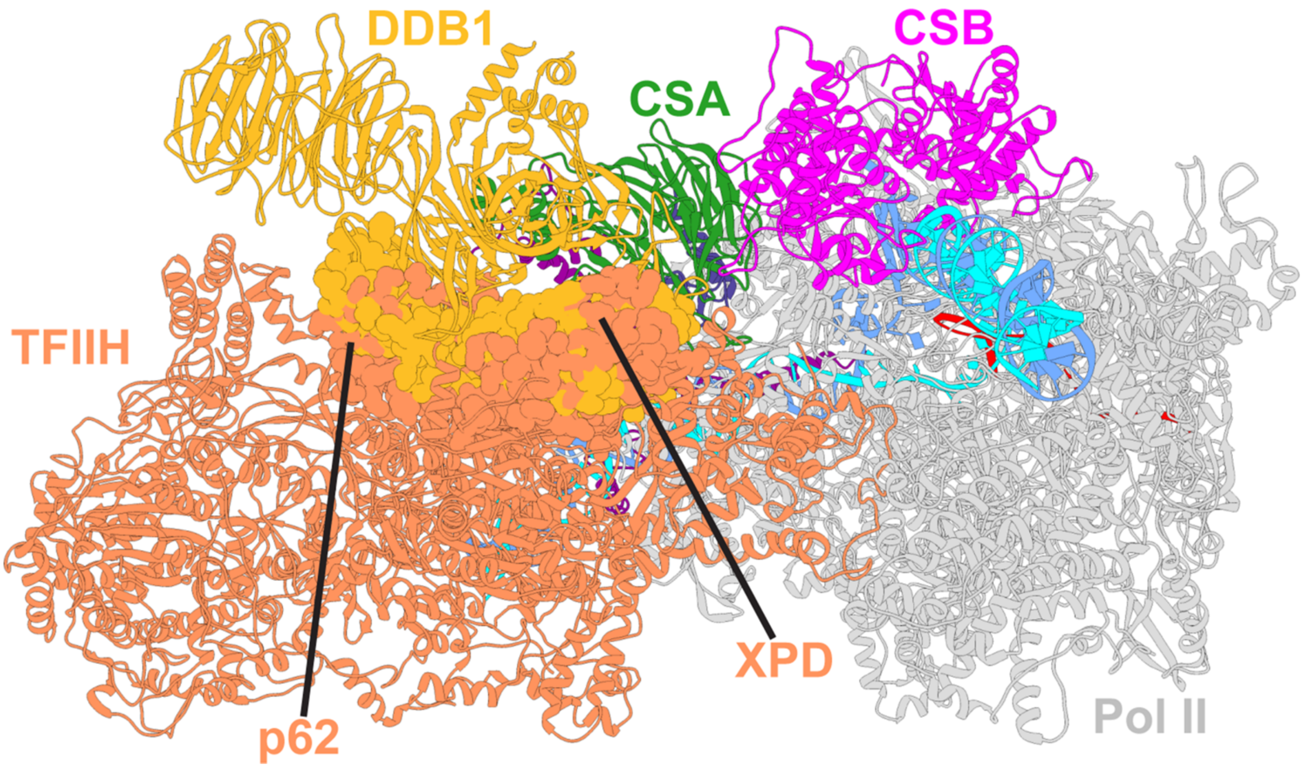

Supplementary Figure 3. ELOF1 orients CSA and DDB1 to prevent clashes with the incoming TFIIH. Overlay of the TCR–CSA–DDB1–VHS complex (PDB ID: 7OOP) and the incoming TFIIH in absence of ELOF1. Steric clashes between TFIIH and core TCR proteins are shown explicitly in van-der-Waals representation. The color-coding scheme is as follows: CSB in magenta, CSA in dark green, DDB1 in gold, UVSSA in purple, and Pol II in gray.

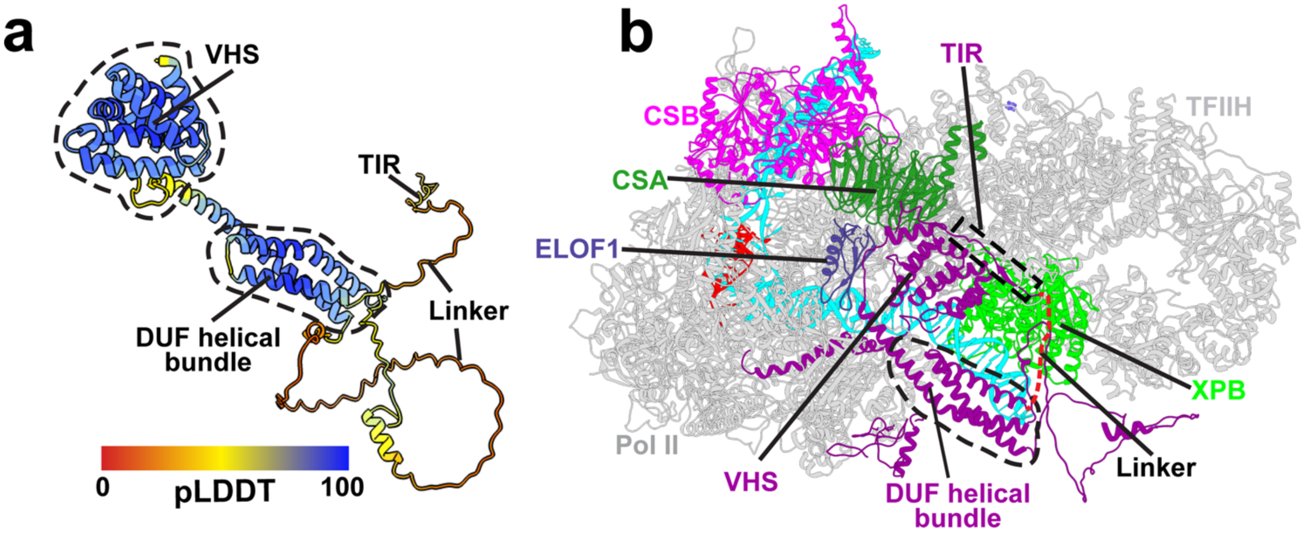

Supplementary Figure 4. The structure of UVSSA modeled by AlphaFold2 and its placement in the overall TCR-TFIIH complex. a, AlphaFold2-predicted structure of UVSSA is shown in cartoon representation with the pLDDT confidence score mapped onto the model and color-coded from red (low confidence) to blue (high confidence). The VHS domain and DUF helical bundle are outlined with black dashed lines. The linker region connecting the DUF helical bundle to the TIR is labeled. **b,** Placement of UVSSA and the DUF helical bundle within the TCR-TFIIH complex. XPB is shown in green, CSB in magenta, CSA in dark green, UVSSA in purple, ELOF1 in violet and Pol II in gray. UVSSA's TIR is outlined with a rectangular dashed box. A red dash line indicates the span of the linker region.

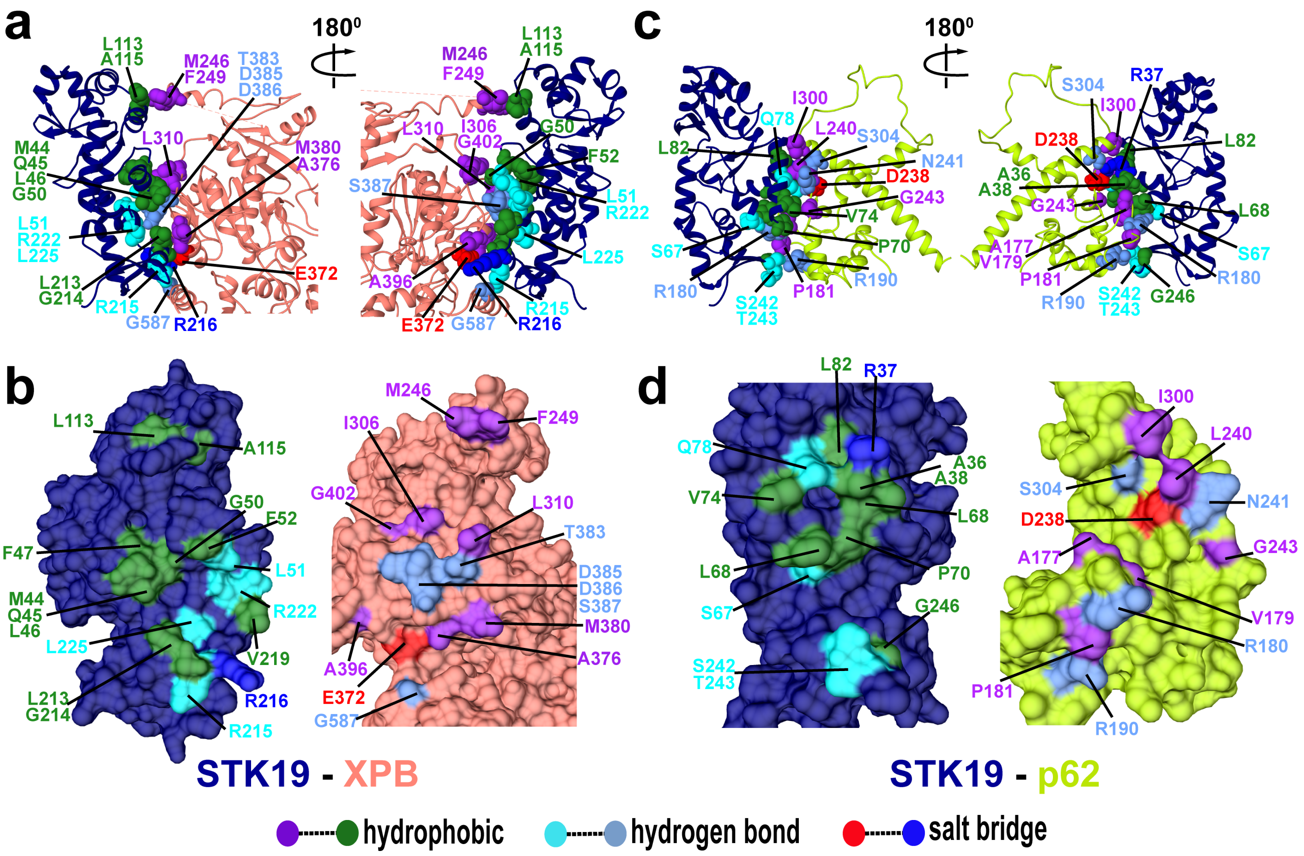

Supplementary Figure 5. STK19 forms extensive interaction interfaces with both XPB and p62. **a,** View of the STK19 with XPB in cartoon representation. Contacting residues are shown in spheres. STK19 is colored in blue and XPB in pink. **b**, Residue contacts mapped onto the STK19 and XPB surface. **c,** View of the STK19 with p62 in cartoon representation. Contacting residues are shown in spheres. STK19 is colored in blue and p62 in green. **d,** Residue contacts mapped onto the STK19 and p62 surface. The side chains of contact forming residues are shown with salt-bridge interactions in red and blue, hydrogen bonding interactions between polar residues in cyan and light blue, and hydrophobic interactions in green and purple.

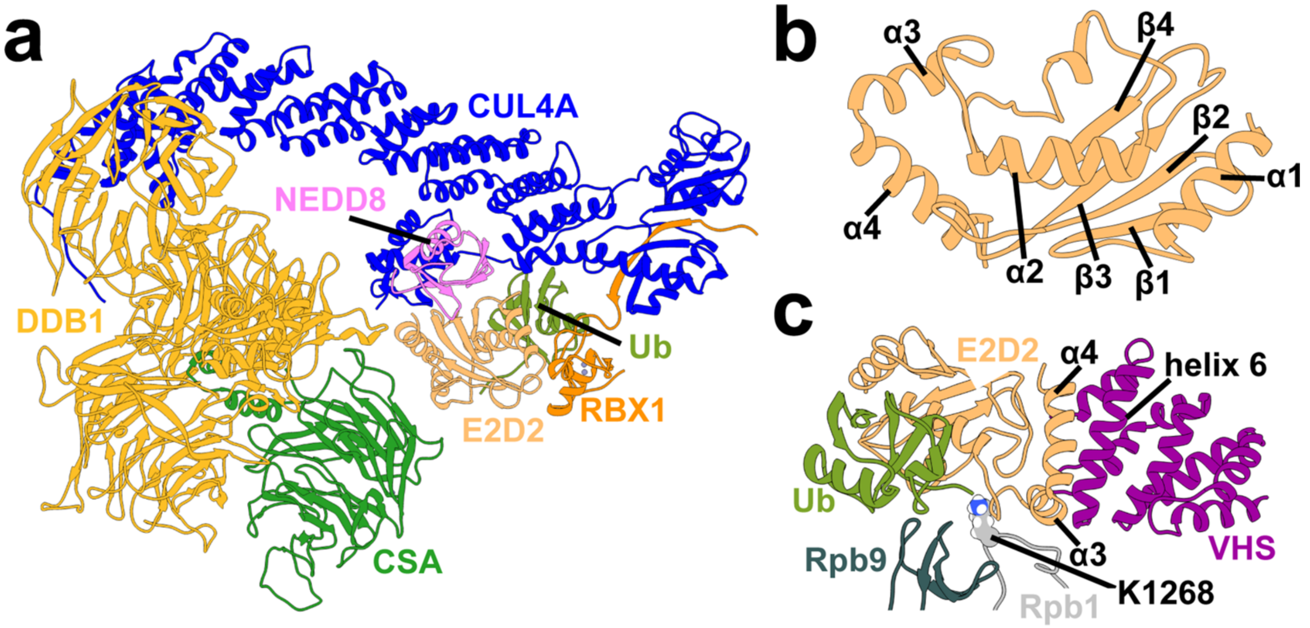

Supplementary Figure 6. Integrative structural model of the CRL4^{CSA} complex. a) CRL4^{CSA} model based on the cryo-EM structure (PDB ID: 8B3I) shown in cartoon representation and colored by protein chain. **b**, Zoomed-in view of the E2D2 enzyme with all α-helices and β-strands labelled. **c**, Zoomed-in view of the E2D2–VHS interface showing the position of Ub near the K1268 ubiquitination site on Rpb1.

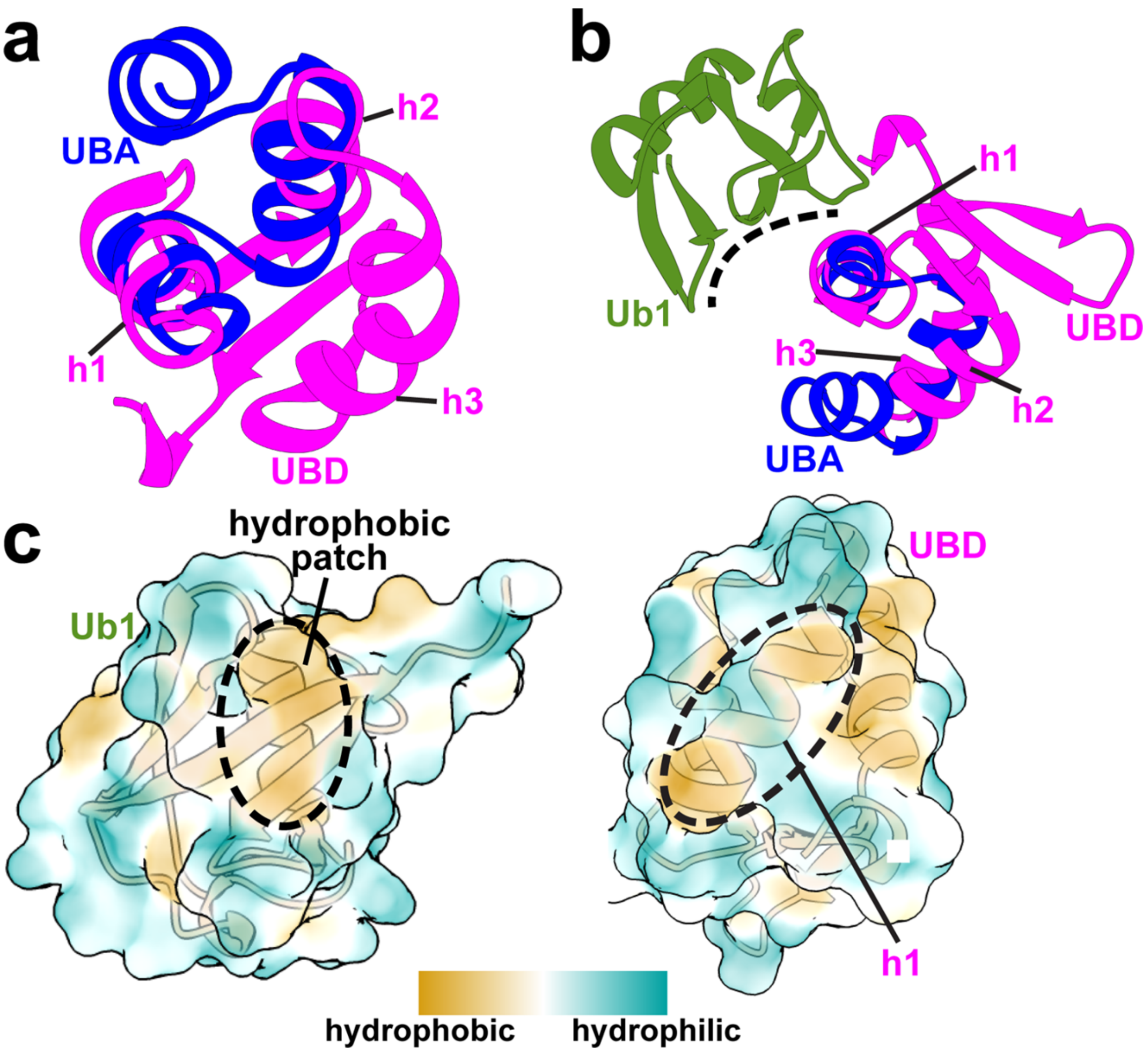

Supplementary Figure 7. Conserved binding modes of UBA and UBD with ubiquitin. a, Structural superposition of UBA (blue) and UBD (purple) shows two conserved helices (h1 and h2) that are superimposable despite the different domain folds. **b**, Depiction of the binding interactions between UBA and UBD with ubiquitin. **c**, UBD–Ub binding interface, shown in cartoon and surface representations with hydrophobic (tan) and hydrophilic (cyan) regions indicated.

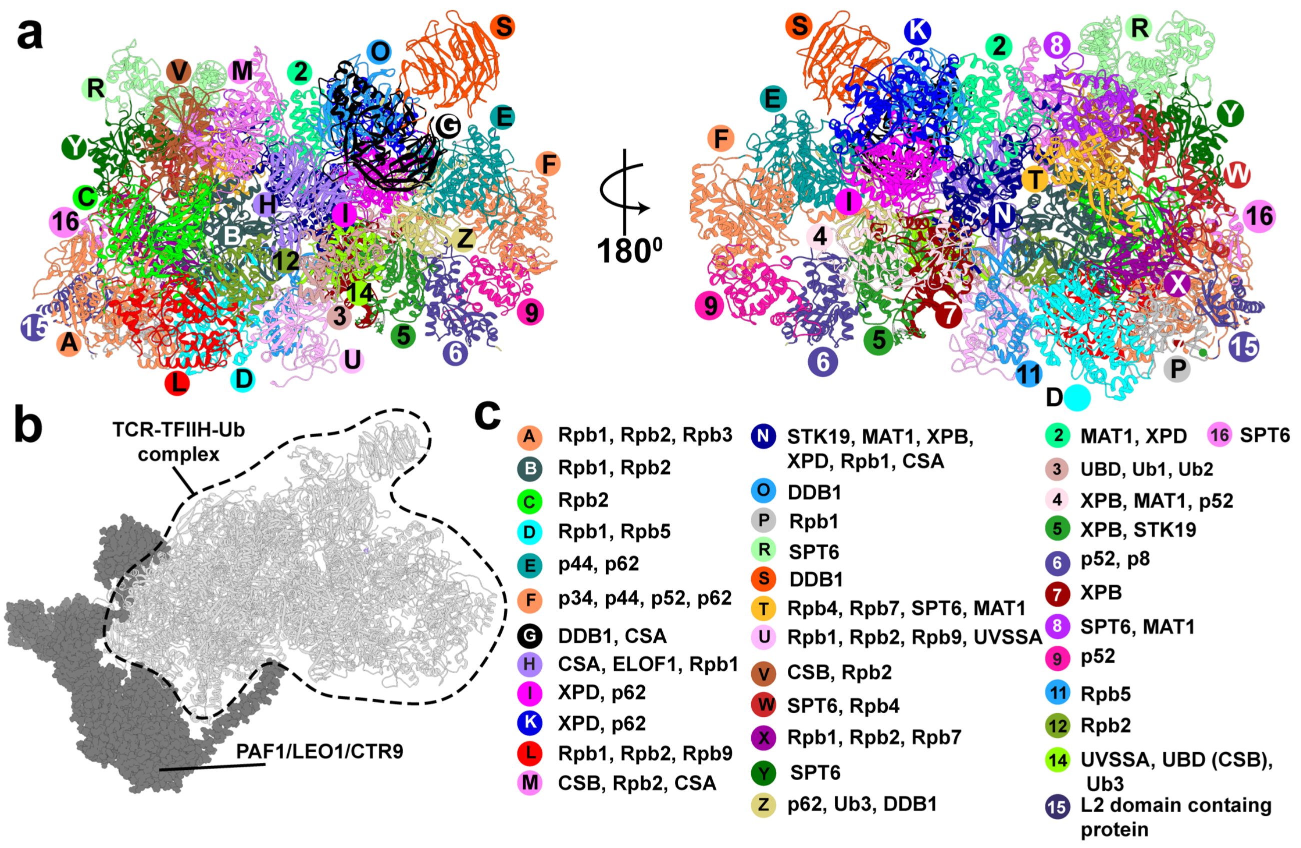

Supplementary Figure 8. Complete set of dynamic communities in the TCR–TFIIH–Ub assembly. a, Complete set of communities identified from dynamic network analysis of the TRC–TFIIH–Ub complex. The complex is shown in anterior and posterior view with communities color-coded and labelled. **b,** Dynamic network analysis was carried out for TRC–TFIIH–Ub (light gray) and the PAF1, LEO1 and CTR9 (dark gray) which were not included in further analysis. **c,** Labels identifying the domains or structural elements participating in each dynamic community.

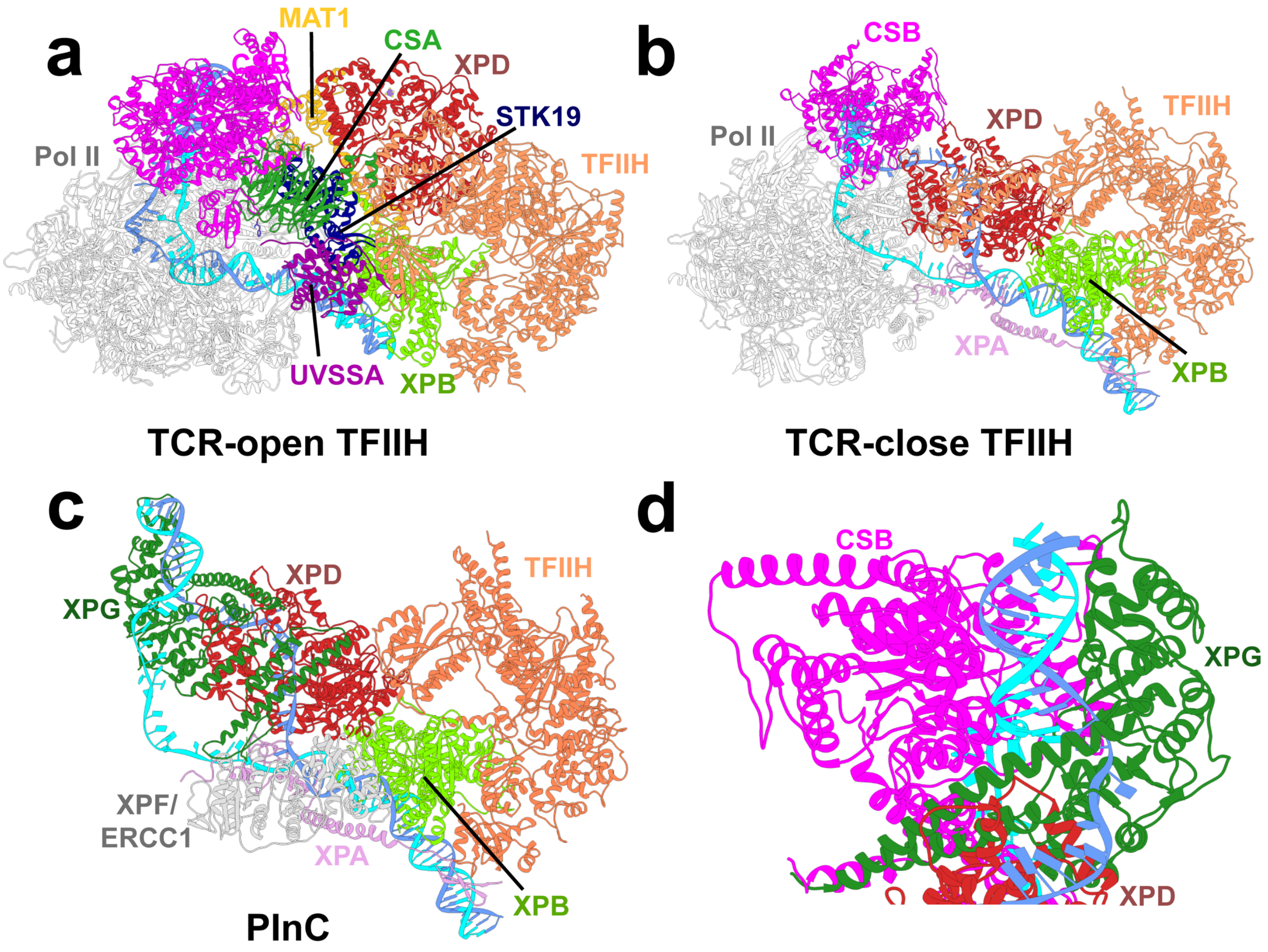

Supplementary Figure 9. Proposed model for the progression of the TCR machinery from an open repair-inhibited conformation to a closed repair-competent state, which precedes stand incision. a, Integrative structural model of the TCR-TFIIH complex with TFIIH subunits and core TCR factors color-coded and labeled. **b,** Integrative structural model of a closed TCR-TFIIH complex resulting from the collapse of the MAT1/Pol II interface upon XPA binding to the expanded DNA bubble. The conformational transition triggered by the MAT1 displacement and STK19 destabilization causes XPD to move toward the XPB and Rpb1 and engage the template stand. This movement also disrupts CSA and UVSSA binding. **c,** Integrative structural model of the pre-incision complex (PInC) in NER. **d**, Structure overlay of the closed-TFIIH and PInC complexes shows XPG and CSB occupying opposite positions on the upstream DNA duplex and competing for dsDNA binding. XPG outcompeting CSB could result in Pol II dissociation from the TRC machinery. The color-coding scheme is as follows: TFIIH is colored in orange, CSB in magenta, CSA in green, UVSSA in purple, STK19 in dark bluet, Pol II, XPF and ERCC1 in gray; MAT1 in light brown, XPD in dark red; XPB in light green; XPA in plum, XPG in dark green.

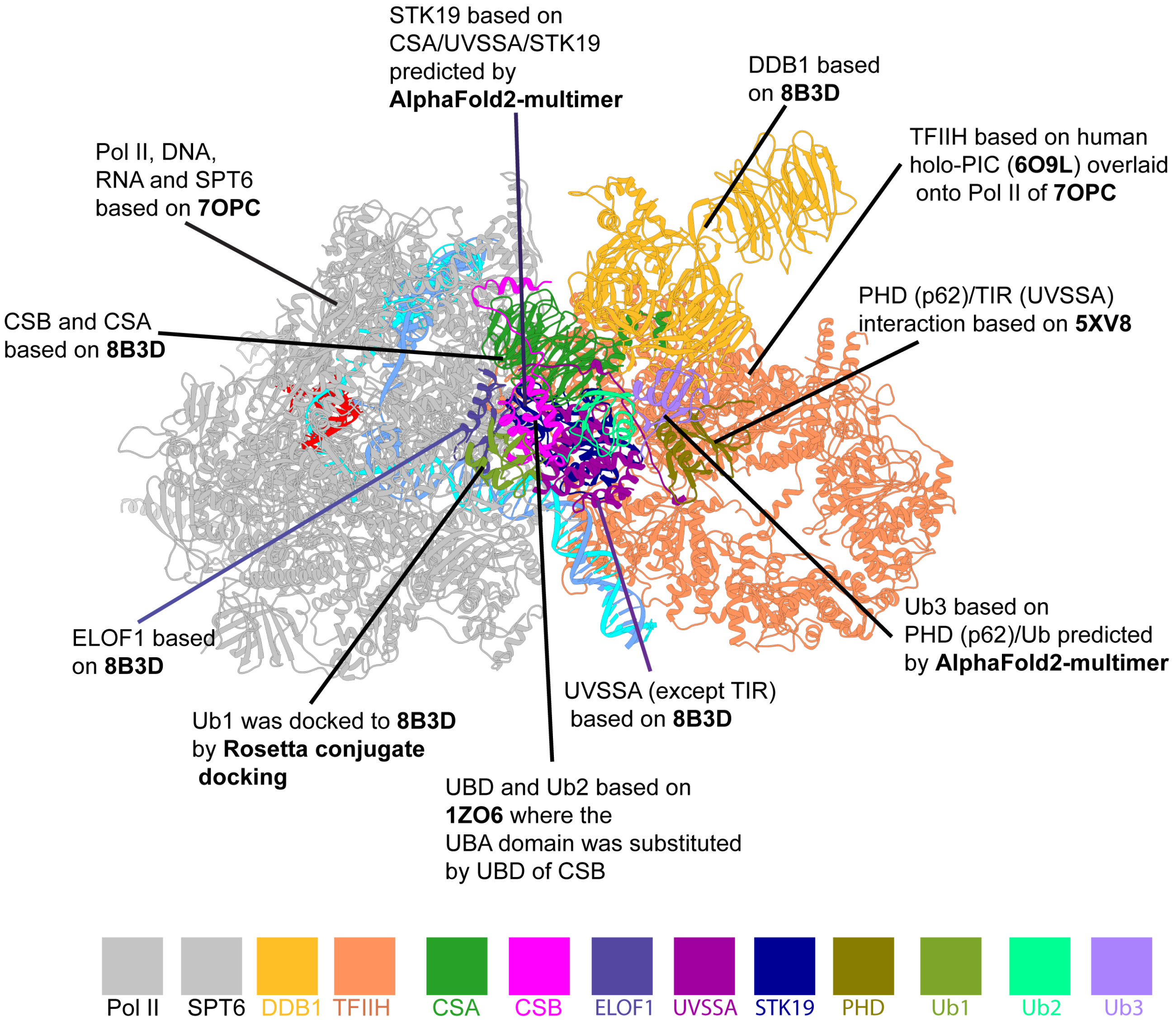

Supplementary Figure 10. Sources of experimental structural information used in constructing the integrative model.

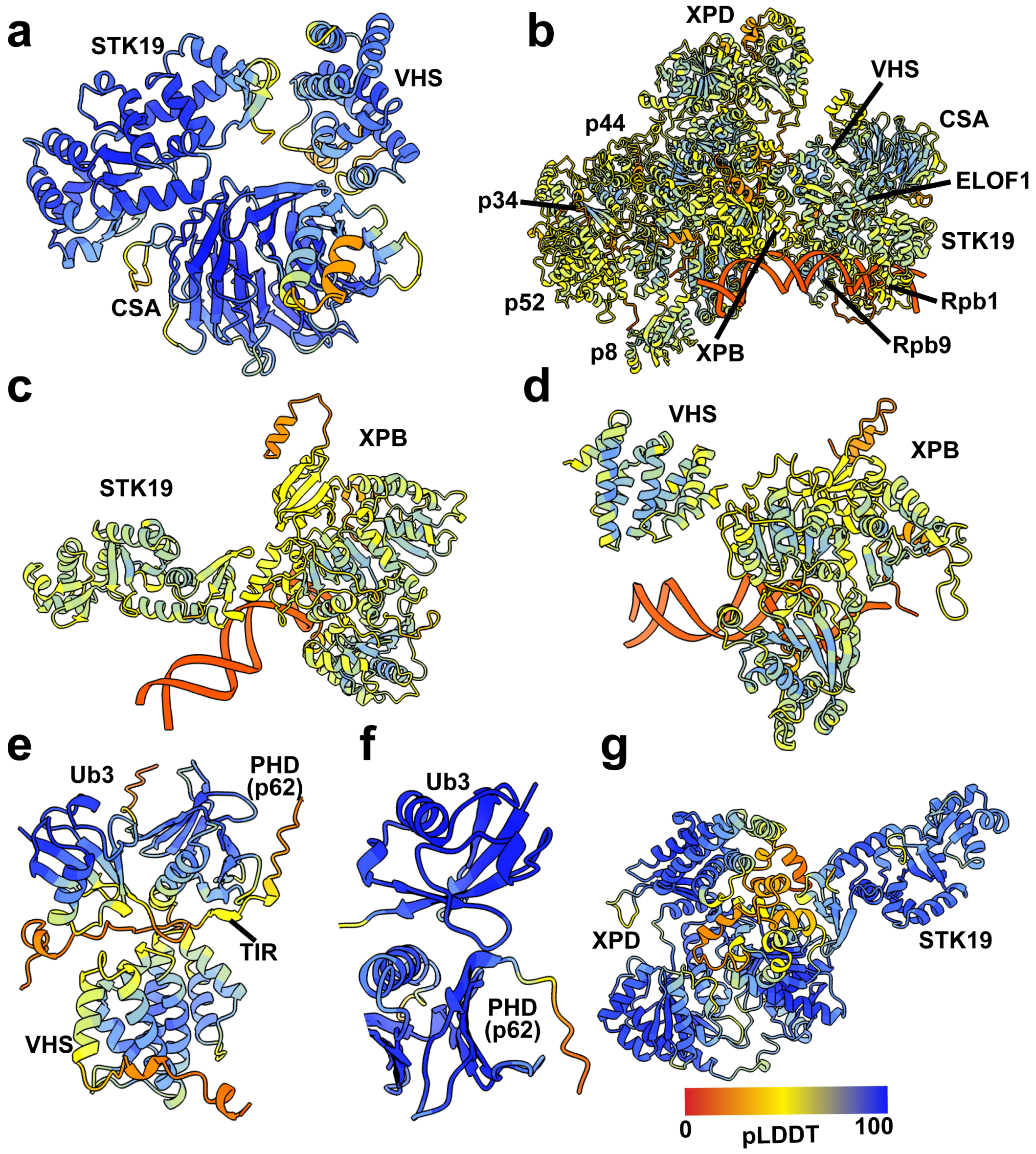

Supplementary Figure 11. Structural models of complexes in this study created by AlphaFold2 and AlphaFold3-multimer. **a**, Complex comprised of CSA, VHS, and STK19 generated with AlphaFold2. **b**, Large-scale complex comprised of XPB, XPD, p62, p52, p44, p34, p8, CSA, VHS, ELOF1, STK19, the clamp head (Rpb1), Rpb9, and DNA that was generated with AlphaFold3. Close-up views of the **c**, STK19–XPB interface and **d**, XPB–VHS (UVSSA) interface, as predicted by AlphaFold3 within the multimer complex depicted in (b). **e**, Complex composed of the UVSSA VHS domain, p62's PHD, Ub3, and the TIR of UVSSA generated with AlphaFold2. **f**, Zoomed-in view of the predicted Ub3–PHD interface. **g**, STK19–XPD complex predicted with AlphaFold2 (but not by AlphaFold3**).** pLDDT confidence score are mapped onto all model and color-coded from red (low confidence) to blue (high confidence).

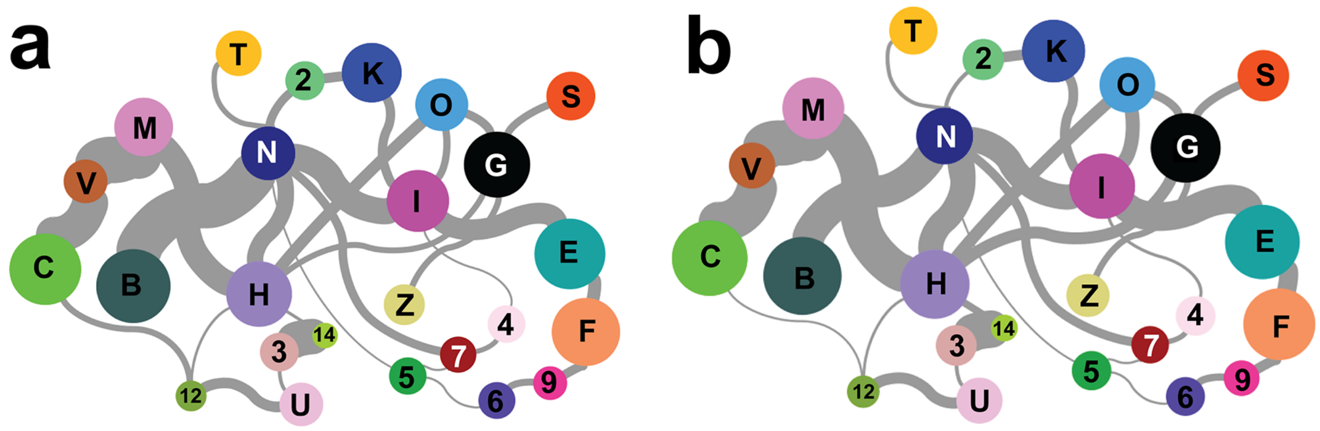

Supplementary Figure 12. Network graphs of the TCR-TFIIH complex obtained by averaging over a) the first or b) the second half of the trajectory frames. Network graphs and dynamic community splittings showed little change if analyzed over a subset of trajectory frames.

Supplementary Table 1: List of simulated systems with corresponding simulation box dimensions, total number of atoms and number of water molecules.

Simulated Systems	Simulation box dimensions (Å)			Total number of atoms	Number of water molecules
	X	Y	Z		
TCR–DDB1	240	261	318	1,882,605	576,781
TCR–DDB1–VHS	247	249	331	1,920,257	587,390
TCR–CSA–DDB1–VHS–ELOF1	240	260	318	1,882,458	576,405
TCR–CRL4^{CSA} (Linear conformation)	288	238	333	2,168,339	665,540
TCR–CRL4^{CSA} (Hinged conformation)	245	268	326	2,027,819	618,788
TCR–CRL4^{CSA} (Twisted conformation)	245	273	329	2,079,547	636,002
TCR–CRL4^{CSA} (with ELOF1)	258	255	331	2,050,219	624,281
TCR–TFIIH complex	285	262	343	2,426,816	735,882
TCR–TFIIH–Ub complex	285	262	343	2,427,784	735,826
