# Supplementary material for: Molecular model of TFIIH recruitment to the transcription-coupled repair machinery
Source: Nat Commun. 2025 Mar 8;16:2341. doi: 10.1038/s41467-025-57593-0 (PMC11890784; doi:10.1038/s41467-025-57593-0)
Supplement: Supplementary file 3 — Description of Additional Supplementary Files [file 41467_2025_57593_MOESM3_ESM.docx]

**Description of Additional Supplementary Files**

**File name:** Supplementary Movie 1

**Description:** Architecture of the TCR–TFIIH complex and interfaces with core TCR proteins that are key for the assembly’s structural integrity. The TCR–TFIIH assembly is shown in cartoon representation with core TCR proteins color-coded and labelled. Zoomed-in views of STK19 and the interfaces of STK19 with core TCR proteins and TFIIH subunits are shown in surface and cartoon representation for: Rpb1 clamp head of Pol II, CSA, the UVSSA VHS domain, XPB, the RING domain of MAT1, XPD and p62.

**File name:** Supplementary Movie 2

**Description:**

Optimal path for the CRL4CSA Cullin4A arm rotation computed using the partial nudged elastic band method. Views of the CRL4CSA complex in 1) the linear, 2) hinged, and 3) twisted BPB domain conformations. The highly directional movement of the Cullin4A arm enables Rpb1 and CSB ubiquitination. Magnified views of the E2D2, NEDD8 and Ub1 near the K1268 ubiquitination site of Rpb1 in the absence and presence of ELOF1. The color-coding scheme follows Fig. 5.

**File name:** Supplementary Movie 3

**Description:** Dynamic communities from network analysis of the TCR–TFIIH complex. The TCR–TFIIH complex is shown in cartoon representation with dynamic communities color-coded as in Fig. 7 and labelled. Zoomed-in views are shown of 1) a cluster of dynamic communities interacting strongly with CSB, CSA and ELOF1, 2) a cluster of dynamic communities surrounding STK19, and 3) a cluster of dynamic communities involving ubiquitin moieties Ub1, Ub2 and Ub3, CSB’s UBD, and the PHD of p62.

**File name:** Supplementary Data 1

Description: Initial and final structures from the molecular dynamics simulations
